# Prognostic Value of the Brixia Radiological Score in COVID-19 Patients: A Retrospective Study from Romania

**DOI:** 10.3390/tropicalmed10050130

**Published:** 2025-05-12

**Authors:** George-Cosmin Popovici, Costinela-Valerica Georgescu, Alina Condratovici Plesea, Anca-Adriana Arbune, Gutu Cristian, Manuela Arbune

**Affiliations:** 1 School for Doctoral Studies in Biomedical Sciences, “Dunarea de Jos” University from Galati, 800008 Galati, Romania; popovicigeorge1989@gmail.com; 2 Pneumophtiziology Hospital Galati, 800189 Galati, Romania; 3 Pharmaceutical Sciences Department, “Dunarea de Jos” University from Galati, 800008 Galati, Romania; 4 Gynecology and Obstetrics Clinic Hospital Galati, 544886 Galati, Romania; 5 Medical Department, “Dunarea de Jos” University from Galati, 800008 Galati, Romania; alina.plesea@ugal.ro; 6 Neurology Department Clinic Institute Fundeni Bucharest, 022328 Bucharest, Romania; anca.arbune@icfundeni.ro; 7 Multidisciplinary Integrated Center for Dermatological Interface Research, 800010 Galati, Romania; 8 “Dr. Aristide Serfioti” Military Emergency Hospital, 800008 Galati, Romania; cristian.gutu@ugal.ro; 9 Medical Clinic Department, “Dunarea de Jos” University from Galati, 800008 Galati, Romania; manuela.arbune@ugal.ro; 10 Infectious Diseases Clinic I, Infectious Diseases Clinic Hospital Galati, 800179 Galati, Romania

**Keywords:** COVID-19, Brixia score, predictor death, emerging infectious disease

## Abstract

The novel coronavirus pandemic, SARS-CoV-2, has a variable clinical spectrum, ranging from asymptomatic to critical forms. High mortality and morbidity rates have been associated with risk factors such as comorbidities, age, sex, and virulence factors specific to viral variants. Material and Methods: We retrospectively evaluated imaging characteristics using the Brixia radiological score in relation to favorable or unfavorable outcomes in adult patients. We included COVID-19 cases, admitted between 2020 and 2022, in a specialized pulmonology hospital with no intensive care unit. We analyzed 380 virologically confirmed COVID-19 cases, with a mean age of 52.8 ± 13.02 years. The mean Brixia radiological score at admission was 5.13 ± 3.56, reflecting predominantly mild-to-moderate pulmonary involvement. Multivariate analysis highlighted the utility of this score as a predictive marker for COVID-19 prognosis, with values >5 correlating with other severity biomarkers, NEWS-2 scores, and a lack of vaccination and hospitalization delay of more than 6 days from symptom onset. Summarizing, the Brixia score is itself an effective tool for screening COVID-19 cases at risk of death for early recognition of clinical deterioration and for decisions regarding appropriate care settings. Promoting vaccination can reduce the severity of radiological lesions, thereby decreasing the risk of death. Technologies based on artificial intelligence could optimize diagnosis and management decisions.

## 1. Introduction

The first case of COVID-19 was confirmed in December 2019 in Wuhan, China, and rapidly spread worldwide. In Romania, the first case was reported on 26 February 2020, in the western region of the country, while in Galați County, located on Romania’s eastern border, it was reported on 7 March 2020.

At the beginning of the pandemic, in the absence of a vaccine, with the unavailability of specific antiviral therapy and limited access to viral diagnostic tests, case management relied largely on clinical data and biological markers. The nonspecific initial manifestations of the disease, most commonly fever, dry cough, and fatigue, could progress through various clinical scenarios, ranging from self-limiting forms to severe cases and death. It was observed that several factors—including demographic characteristics, comorbidities, immunity, and access to testing and early treatment, especially in the context of overwhelmed healthcare services—significantly influenced the severity of COVID-19 and, consequently, the prognosis of the infection [1,2].

The biomarkers frequently recommended for COVID-19 assessment include elevated levels of C-reactive protein and interleukin-6, increased coagulation markers, elevated liver enzymes, troponin, or creatine kinase, progressive lymphocyte depletion, and an increase in neutrophil count and the neutrophil-to-lymphocyte ratio (NLR) [3,4].

The reference imaging method for COVID-19 assessment studies is computed tomography (CT), but other methods, such as chest X-ray and ultrasound, have also proven useful. Chest X-ray has been used for screening and monitoring patients in China, Italy, and the United Kingdom. Radiological abnormalities are like those described on CT, with the presence of ground-glass opacities and bilateral peripheral consolidations. These abnormalities reach peak intensity 10–12 days after symptom onset on chest X-ray, compared to 6–11 days on CT imaging [4]. Although CT sensitivity is superior to initial chest X-ray (86.2–98% vs. 59.1–69%), chest X-ray offers advantages such as shorter processing and decontamination times, as well as broader accessibility in most medical facilities worldwide. Thus, chest X-ray can confirm the diagnosis of COVID-19 and monitor disease severity while avoiding unnecessary CT exposure and helping to triage cases that require CT examination [5,6].

The interpretation of radiological images in COVID-19 is based on algorithms that generate lesion scores. Additionally, algorithms based on neural network architectures, combined with advanced artificial intelligence techniques integrated into radiological imaging, have also been developed [7,8].

We hypothesize that higher Brixia radiological scores are associated with increased mortality and more severe clinical outcomes in patients with COVID-19. The objective of this study is to assess the prognostic value of the Brixia score in evaluating disease severity among hospitalized Romanian patients during the COVID-19 pandemic, in relation to their demographic, clinical, and laboratory characteristics.

## 2. Materials and Methods

We conducted a retrospective cohort study between March 2020 and December 2022 at the Galați Pneumophthisiology Hospital, located in the southeastern border region of Romania. The study included adult patients over 18 years old who were confirmed to have SARS-CoV-2 infection through a positive RT-PCR test from nasopharyngeal secretions. Patients unable to sign the regular informed consent were excluded.

We collected demographic data (age, sex, living environment, and education level), health risk factors (smoking, alcohol consumption, and occupational exposure to toxins), SARS-CoV-2 vaccination status (at least one vaccine dose), and clinical, radiological, and laboratory data. We also recorded associated chronic comorbidities, including hypertension and cardiovascular, neurological, pulmonary, hepatic, renal, or metabolic diseases.

We recorded the clinical manifestations of COVID-19, categorizing them as 0 or 1 based on the symptoms reported by patients from the onset of the disease until hospital admission. Treatment was administered according to local protocols based on COVID-19 severity, but it is not analyzed in this study [9,10,11,12].

Chest X-rays were performed according to standard protocols using posteroanterior or anteroposterior projections, depending on the clinical condition of the patients. The X-rays were interpreted and classified as either normal or abnormal by two independent examiners, with discrepancies reviewed by a pulmonologist in the context of the clinical presentation. Abnormal X-rays were further analyzed based on the anatomical distribution of pulmonary involvement—right or left lung, upper, middle, or lower zones, and perihilar or lateral regions. Additional radiological features, such as the presence of pleural effusions and pulmonary nodules, were also documented [13].

**Figure 1:**
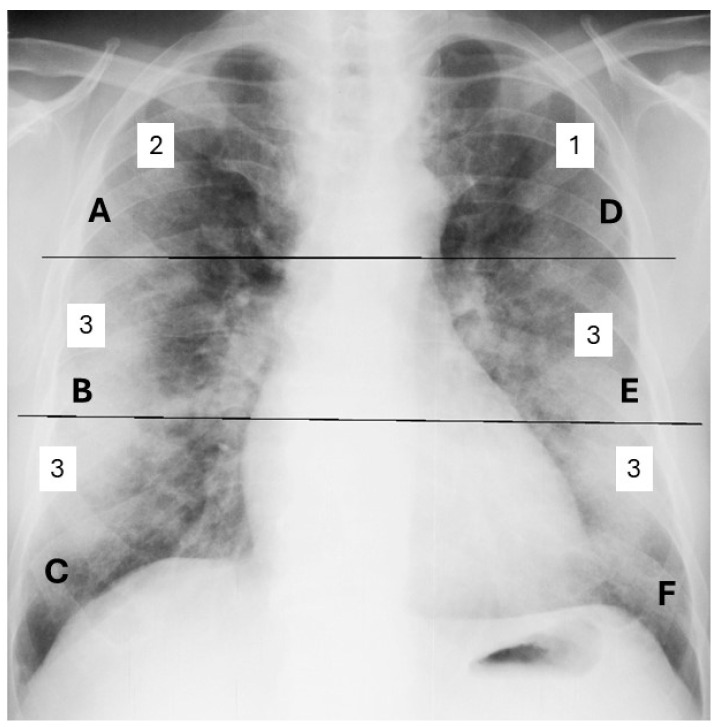
Brixia scoring by the sum of assigned scores from 0 to 3 for each of the 6 regions of the lungs. (A, B, C, D, E, F). Example of a global Brixia score of 15.

The severity of lung involvement was assessed using the Brixia scoring system, which quantifies radiological lung damage in COVID-19 infection. This algorithm divides the lungs into six regions, each evaluated individually and assigned a score from 0 to 3 based on the severity of the observed lesions. The total score is obtained by summing the scores from all regions, providing a clear overall assessment of lung involvement [14,15]. The total Brixia score ranges from 0 (indicating no pleural or pulmonary vascular changes) to 18 (reflecting the most severe lung involvement) (Figure 1). Other radiological abnormalities, such as pleural effusions, are not included in the Brixia score and were therefore not considered in the assessment of pulmonary severity [16].

We classified disease severity according to WHO recommendations and recorded complications. Patients were divided into two categories: favorable and unfavorable outcomes. A favorable outcome was defined as improvement and recovery by the time of discharge. An unfavorable outcome was assigned to patients who either died in the hospital or experienced clinical deterioration requiring transfer to intensive care units, where they subsequently died [17].

The comparison of the Brixia score value as a predictor of COVID-19 severity was conducted in relation to the NEWS2 score, which is based on six parameters (respiratory rate, oxygen saturation, systolic blood pressure, pulse rate, level of consciousness or new confusion, and body temperature), each scored from 0 to 3, with an additional 2 points added if supplemental oxygen is required. A NEWS2 score of 6 or higher was considered indicative of clinical deterioration [18,19].

Statistical data analysis was performed using XL-STAT version 2020.1 for quantitative data and calculating mean values, standard deviation (SD), and percentage distribution for each group. The normality of data distribution was assessed using a visual inspection method (histogram). Comparisons were made using the Student’s *t*-test for parametric data and the Mann–Whitney U test for non-parametric data. For categorical variables, the chi-square test was applied, with a *p* significance level of 0.05. Logistic regression was used to predict unfavorable outcomes based on the Brixia score, in comparison with other severity biomarkers of COVID-19. An area under the curve (AUC) analysis was performed to evaluate the predictive performance of the Brixia score for favorable outcomes in COVID-19 patients [20].

This study was performed in accordance with the Declaration of Helsinki and received institutional approval from the Ethics Committee of the Pneumology Hospital Galati (under the number 225/09.01.2024).

## 3. Results

### 3.1. Demographic and Behavioral Data

The study included 380 patients aged between 19 and 91 years, with a median age of 58 years and a mean age of 52.8 ± 13.02 years. Elderly patients over 65 years accounted for 34.4% of cases, while 31.3% of patients were in the 50–60 age group. The male-to-female sex ratio was 1.37, indicating a significantly higher proportion of male patients.

Other demographic characteristics included a predominance of urban residency and a high school education level, though no significant differences were observed between occupational categories. Identified health risk behaviors included smoking (35%), alcohol consumption (25%), and occupational exposure to respiratory hazards (8.68%). Only 16% of patients had received at least one dose of the SARS-CoV-2 vaccine [Table A1].

### 3.2. Clinical and Biological Characteristics of Hospitalized COVID-19 Patients

The spectrum of chronic comorbidities highlighted hypertension as the most common condition (53.42%), followed by obesity (40.26%) and chronic heart diseases (27.63%). Among pulmonary diseases, chronic obstructive pulmonary disease (COPD) was reported in 16.5% of cases, while bronchial asthma was observed in 6.57% of patients.

The clinical presentation of COVID-19 included symptoms with the following frequencies: fever (71.32%, 271 patients), headache (66.58%, 253 patients), anosmia (26.05%, 99 patients), ageusia (26.32%, 100 patients), myalgia (62.37%, 237 patients), arthralgia (41.05%, 156 patients), chest pain (48.68%, 185 patients), abdominal pain (12.37%, 47 patients), nausea/vomiting (6.58%, 25 patients), diarrhea (9.74%, 37 patients), dysphagia (23.68%, 90 patients), rhinorrhea (34.74%, 132 patients), cough (88.16%, 335 patients), respiratory difficulty (55.53%, 211 patients), and hemoptysis (2.89%, 11 patients).

The mean duration of symptoms from onset to hospital admission was 6.28 ± 3.11 days, with a median of 6 days (ranging from 1 to 21 days). Oxygen saturation at admission was below 93% in 52.1% of patients, with recorded values ranging from 55% to 98% and a mean of 88.80 ± 8.07.

The NEWS2 score at the time of hospital admission ranged from 0 to 13, with a mean value of 5.07 ± 3.5. Using a threshold score of 6 to indicate potential clinical deterioration, 43.94% of hospitalized patients with COVID-19 were identified as being at risk.

The most frequently observed biological abnormalities included increased inflammatory markers (CRP, ESR, and fibrinogen), an elevated neutrophil-to-lymphocyte ratio (NLR), and increased levels of LDH, AST, and ALT. Coagulation markers were not consistently available during the study period and were therefore not analyzed in this study [Table A2].

### 3.3. Radiological Characteristics of Hospitalized COVID-19 Patients

The analysis of each of the six pulmonary radiological regions and their partial scores revealed the most severe involvement in the lower lung regions, with a progressive cranio-caudal increase in score values. The overall distribution of radiological lesions appeared symmetrical, although the number of cases with a score of 3 was slightly higher in the right lung regions (18.6%) compared to the left lung (15%). Additionally, severe involvement of the upper lung region was more frequently observed in the right lung (5.7%) than in the left side (3.6%) (Table 1).

The global radiological score (Brixia) assessed at admission ranged from 0 to 18, indicating variability in the severity of pulmonary radiological lesions. At admission, the mean score was 5.13 ± 3.56, with a median of 5, reflecting mild-to-moderate pulmonary involvement in most patients.

### 3.4. Disease Progression and Complications

According to severity criteria, the clinical progression of COVID-19 infection was classified as follows: 22.11% (84 patients) presented with mild forms; 67.89% (258 patients) had moderate forms; and 10% (38 patients) developed severe forms.

Complications were recorded in 33.42% (127/380) of patients and were classified as follows: respiratory complications (thirty-three cases), cardiovascular complications (nine cases), gastrointestinal complications (eighty-one cases), renal complications (two cases), septic complications (one case) and allergic complications (one case).

Hepatitis associated with SARS-CoV-2 infection occurred in 15.9% of cases. Acute surgical abdomen, requiring surgical intervention, was caused by mesenteric ischemia. Severe respiratory dysfunction complicated 7.9% of cases, making it the most frequent reason for patient transfer. Other respiratory complications included pleural effusion and lung abscess. One patient was newly diagnosed with lung cancer during investigations for COVID-19. The most severe complications were cardiovascular events, leading to cardiopulmonary arrest in the context of thromboembolic lesions affecting the heart, lungs, or brain. However, no autopsy confirmation was available. Cerebrovascular accidents occurred in three patients: one experienced a transient ischemic attack and later improved, while two developed severe neurological deficits and coma, requiring transfer to intensive care units. Other reported complications included acute kidney failure in two patients, one case of sepsis, and three newly diagnosed cases of diabetes mellitus.

Most patients (94.5%) were discharged in an improved or fully recovered state. In-hospital mortality was 1.3% (5 patients), with deaths caused by cardiorespiratory arrest. An additional 4.2% of patients died after being transferred in a deteriorated condition to intensive care units in other hospitals or medical facilities.

### 3.5. Correlations Between Favorable and Unfavorable Outcomes

No statistically significant associations were found between disease progression and smoking or comorbidities such as obesity, chronic pulmonary diseases, or cardiovascular diseases. However, an unfavorable outcome was significantly associated with age over 65 years, male sex, less than 8 years of formal education, alcohol consumption, delayed hospitalization (more than 6 days after symptom onset), presence of respiratory distress and chest pain, increased CRP levels, a NLR of 4 or higher, and a Brixia radiological score greater than 5 (Table 2).

**Figure 2:**
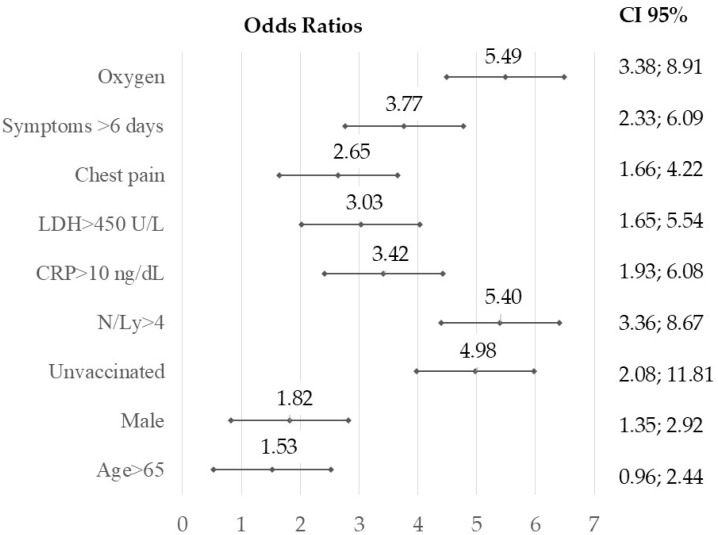
Forest plot illustrating the odds ratios and 95% confidence intervals from multivariate logistic regression to identify the relationship between a Brixia score over 5 and other predictors of unfavorable COVID-19 outcomes: unvaccinated, late presentation after 6 days from the onset, necessity of oxygen supply, increased CRP (>10 ng/dL), LDH > 450 U/L, and NLR over 4; level of significance *p* < 0.01, two-tailed.

The Brixia score was higher in unvaccinated patients compared to vaccinated patients (4.12 vs. 1.75, *p* < 0.001). Multivariate analysis highlighted the utility of this score as a predictive marker for COVID-19 prognosis. Brixia scores > 5 were correlated with other biological severity markers, such as the neutrophil-to-lymphocyte ratio (N/Ly), CRP, and LDH levels, as well as with the lack of vaccination, delayed hospitalization beyond six days from symptom onset, and the need for oxygen therapy (Figure 2).

A statistical analysis of severity scores demonstrated that a Brixia score > 5 and a NEWS score > 6 are concordant and independent predictors of unfavorable clinical outcomes (OR = 8.82; 95% CI: 5.35–14.53; *p* < 0.001).

**Figure 3:**
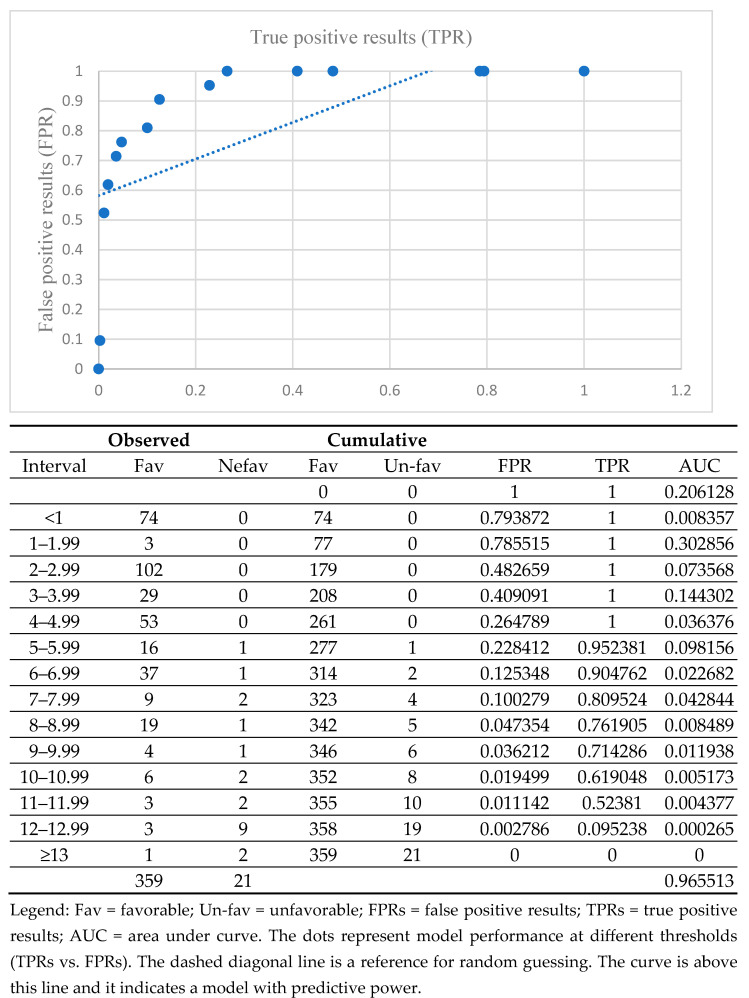
AUC for various cut-off Brixia scores in differentiating between favorable outcomes due to COVID-19 and unfavorable outcomes.

The analysis of the Brixia score as a prognostic marker for COVID-19 progression, based on the AUC calculation, indicated a value of 0.96, demonstrating a high overall performance of this test in identifying cases with an unfavorable outcome (Figure 3 with additional table).

## 4. Discussion

The magnitude of the COVID-19 pandemic and the high rate of severe disease progression from its onset have led to numerous studies and analyses aimed at identifying risk factors and severity markers and developing predictive scores for severe infection outcomes. Although significant progress has been made in understanding the pathogenic mechanisms of SARS-CoV-2 infection, the virus’s evolving virulence and the pathogenic differences arising from its interaction with the human host result in variable study outcomes in the medical literature, depending on the population and pandemic waves [21].

### 4.1. Behavioral and Clinical Characteristics of COVID-19 Within the Study Population

In our study, alcohol consumption was associated with an unfavorable outcome in COVID-19 patients. However, at the beginning of the pandemic, alcohol was erroneously promoted as having a protective effect against SARS-CoV-2 infection, contributing—along with isolation measures and lockdowns—to an alarming increase in alcohol intoxication cases [22,23].

Smoking was linked to severe disease progression, contradicting a controversial early pandemic study that suggested cigarette smoke might have a protective effect against SARS-CoV-2 infection. Although this hypothesis has been disproven by numerous studies, the role of smoking in COVID-19 remains a topic of debate [24,25].

Obesity was the most prevalent behavioral risk factor among the patients in our study, affecting 40.26% of cases. However, it did not significantly influence disease severity, despite the World Health Organization (WHO) classifying obesity as a major risk factor for severe COVID-19. The link between obesity and severe disease progression has been explained through physiological, biochemical, immunological, and anatomical factors [17].

The frequency of symptoms in our study aligns with most published medical reports, with cough, fever, headache, chest pain, arthralgia, and dyspnea being the most common. Meanwhile, anosmia and ageusia were reported less frequently. Although hemoptysis is not considered a characteristic symptom of COVID-19, it was observed in nine cases in our study without tuberculosis association, and its etiology remains unclear [26].

### 4.2. The Role of the Brixia Score in Predicting COVID-19 Severity

Studies conducted early in the pandemic, when access to SARS-CoV-2 testing was limited, highlighted the role of the semi-quantitative Brixia chest X-ray (CXR) score in COVID-19 diagnosis and prognosis, particularly when combined with epidemiological data and characteristic symptoms of infection [27].

Correlations were observed between the Brixia score, symptom duration from onset, and CRP levels, with the sensitivity of radiological lesions as a marker of COVID-19 progression estimated at 61.1% and 68.1% [28,29].

The Brixia severity score was associated with intubation, the need for non-invasive ventilation, and mortality, with a cutoff value of 6 demonstrating a sensitivity of 77% and a specificity of 73% in predicting the need for intubation [30].

Our study results align with the correlations reported in the medical literature between the Brixia score and other severity biomarkers such as NLR, CRP, and LDH. Furthermore, the AUC analysis supports the use of the Brixia score as a reliable marker for identifying patients at risk of death, enabling early intervention, timely admission to intensive care units equipped with advanced medical technology, and prompt initiation of life-support measures.

A study conducted in Sarajevo in 2021 highlighted the impact of CRP on the Brixia score, confirming its predictive value [31]. Additional variables with predictive potential for the Brixia score included male sex, a lack of COVID-19 vaccination, lactate dehydrogenase (LDH), albumin levels, and severe infection [32].

The neutrophil-to-lymphocyte ratio (NLR) is a biomarker of systemic inflammatory status, reflecting the balance between acute and chronic inflammation, and is predictive of mortality even in the general population. Several recent studies have demonstrated that NLR is an independent risk factor for critical illness and in-hospital mortality in COVID-19 patients. In our study, we identified a strong correlation between this marker and the Brixia score [33,34,35].

In logistic regression analysis, the need for non-invasive oxygen supplementation via mask or nasal cannula was not a severity criterion but rather a consequence of cumulative pathogenic mechanisms reflected by inflammatory and coagulation markers. Although LDH has limited diagnostic value as a non-specific enzyme, its correlation with extensive pulmonary lesions in COVID-19 can be explained by the microthromboses accompanying these lesions [36,37].

Compared to the qSOFA, MREMS, and RPAS scores, the NEWS2 score presented the best predictive power for the early recognition of clinical deterioration in patients with COVID-19 and for decisions regarding the appropriate care setting [38].

The NEWS2 scale assessment of the patients with COVID-19 found that a score of 6 or more had good sensitivity (78.1%) and specificity (89.4%) for predicting an aggressive course of disease and poor outcome [39].

The Brixia score positively correlated with NEWS2 and both could be incorporated into a predictive model to predict in-hospital mortality [40].

The median duration of symptoms from onset to hospitalization was six days, with an asymmetric distribution of values reported in our study. Therefore, performing and analyzing chest radiographs at admission does not correspond to the same time interval in relation to the progression of COVID-19 infection. Given that pulmonary radiological changes in COVID-19 may have a latency of 10–12 days, radiological examinations in this study were performed within the first five days from symptom onset in 50% of cases. Consequently, the radiological score may have failed to capture the emergence of certain specific imaging changes [4].

### 4.3. Artificial Intelligence and Brixia Score: Current Insights and Future Directions

Since the onset of the COVID-19 pandemic, machine learning systems have been developed to facilitate patient triage and enhance diagnostic accuracy. While these models have demonstrated promising performance, their generalizability across heterogeneous datasets and varying imaging conditions has proven challenging [41].

A growing body of research has investigated the application of artificial intelligence (AI) in the analysis of COVID-19-related imaging datasets, emphasizing the prognostic relevance of the Brixia score and its correlation with clinical outcomes. Notably, both the admission score and the maximum score recorded during hospitalization have been associated with disease progression [42].

Comparative analyses have shown that AI-derived scores assessing pulmonary involvement exhibit a similar discriminatory capacity to that of the Brixia score assigned by experienced radiologists in predicting the severity of COVID-19. Furthermore, the deployment of AI-based diagnostic tools has demonstrated feasibility even in resource-limited settings [43].

Certain commercially available AI solutions have alleviated diagnostic uncertainty, particularly among medical students, residents, and less experienced radiologists, by supporting the interpretation of subtle findings and mild pneumonia cases [44].

Although AI-generated heatmaps may indicate relevant visual correlations, structural variability and the absence of radiological information in specific pulmonary regions—such as the right lower lobe or the pericardial area—pose quantification challenges and limit interpretability. A recent study on automated severity prediction underscored that chest radiographs obtained at hospital admission may offer limited incremental value over clinical assessment, particularly due to the lack of detectable radiographic alterations in early-stage disease [45].

Nevertheless, prior to widespread clinical integration, further large-scale, prospective studies are warranted to validate the consistency and reliability of these AI-driven approaches [43].

Future analyses of the COVID-19 imaging database could aim to improve the accuracy of radiologic images and integrate information using algorithmic models based on artificial intelligence, applied both during the acute phase of the disease and for monitoring post-COVID-19 syndrome [46,47].

### 4.4. Study Limitations

The patients included in the study were hospitalized in a pulmonology specialty hospital that lacked an intensive care unit. According to the local COVID-19 case management protocol, only non-critical patients were admitted, based on clinical severity criteria. The reported duration of illness from symptom onset to hospitalization was based on information provided by patients or their caregivers, which may have been subject to recall bias and subjective symptom perception. Additionally, the variable duration from symptom onset to hospitalization when chest radiographs were performed—often within the first week—could have led to an underestimation of the Brixia score. Chest radiographs were not routinely repeated on day 10 from symptom onset in most cases, as the clinical course was favorable. In cases with an unfavorable outcome, patients either experienced sudden death or were transferred to a higher-level facility where they underwent CT examination, but CT severity scores were not available for comparation with radiological scores. External validation of the results is missing. A subgroup analysis comparing Brixia score sensitivity in patients imaged before versus after day 6 was not available but will be considered in future studies. This study was conducted during the early waves of the COVID-19 pandemic. Genetic testing of SARS-CoV-2 variants was not available, preventing a differentiated analysis of the Brixia score based on viral variants that may have differed in virulence.

## 5. Conclusions

The Brixia score is an effective tool for screening COVID-19 cases at risk of death during the pandemic. Patients with Brixia > 5 should be prioritized for ICU admission. The Brixia score correlates well with other severity biomarkers. Symptomatic patients hospitalized within the first five days of COVID-19 onset require close clinical and biological monitoring, which may justify repeating chest radiographs in the second week of illness. Future prospective validation and applications of Brixia scoring should be extended for predicting the severity of other lung diseases. The advancement of artificial intelligence-based diagnostic technologies could enhance the accuracy of imaging interpretation, optimizing diagnostic processes and clinical decision-making.

## Figures and Tables

**Table 1:** Partial severity scores of pulmonary radiological lesions (Brixia) at the time of hospital admission for COVID-19 patients.

Anatomy Region	Score	Score 3		Score 2		Score 1		Score 0	
	Average ± SD	*n*	%	*n*	%	*n*	%	*n*	%
A	0.45 ± 0.84	22	5.79%	22	5.79%	63	16.58%	273	71.84%
B	0.88 ± 0.80	16	4.21%	55	14.47%	180	47.63%	129	33.95%
C	1.26 ± 0.85	33	8.68%	105	37.63%	173	45.53%	69	18.68%
D	0.37 ± 0.75	14	3.68%	21	5.52%	58	15.26%	278	75.53%
E	0.86 ± 0.76	12	3.15%	52	13.68%	188	49.47	128	33.68%
F	1.30 ± 0.86	31	8.15%	124	32.63%	156	41.05%	69	18.16%

**Table 2:** Correlation analysis of favorable and unfavorable outcomes in hospitalized COVID-19 patients.

		Favorable	Unfavorable	OR	CI 95%	*p*
Age [years]	>65	119	12	2.68	1.13; 6.36	0.024
	<65	240	9			
Gender	Male	203	17	3.26	1.13; 9.36	0.027
	Female	156	4			
Formal education [years]	>8	225	9	3.26	1.39; 7.66	0.006
	≤8	104	12			
Smoking	Yes	235	12	1.42	0.58; 3.45	0.437
	No	124	9			
Alcohol	Yes	85	10	2.93	1.24; 6.89	0.013
	No	274	11			
Obesity	Yes	147	6	1.73	0.66; 4.52	0.261
	No	212	15			
Time from onset to admission [days]	≥6	175	15	2.62	1.02; 6.71	0.043
	<6	184	6			
Chest pains	Yes	168	17	4.83	1.75; 13.32	0.002
	No	191	4			
Respiratory distress	Yes	152	17	5.78	2.14; 15.64	<0.001
	No	207	4			
NLR	>4	139	20	31.65	8.39; 119.31	<0.001
	≤4	220	1			
CRP [ng/dL]	<10	317	10	8.30	3.75; 18.35	<0.001
	≥10	42	11			
LDH [U/L]	<450	320	13	5.04	2.13; 11.94	<0.001
	≥450	39	8			
Brixia Score > 5	Yes	83	20	66.50	21.30; 207.60	<0.001
	No	276	1			

Legend: COPD—chronic obstructive pulmonary diseases; CCD—chronic cardiac disease; NLR—neutrophil/lymphocyte ratio.

## Data Availability

The raw data supporting the conclusions of this article will be made available by the authors on request.

## Abbreviations

The following abbreviations are used in this manuscript:

ALT	Alanine transaminase
AST	Aspartate aminotransferase
AUC	Area under the curve
CI	Confidence interval
COVID-19	Coronavirus disease 2019
CT	Computed tomography
CCD	Chronic cardiac disease
COPD	Chronic obstructive pulmonary disease
CRP	C-reactive protein
ERS	Erythrocyte sedimentation rate
FPRs	False positive results
LDH	Serum lactate dehydrogenase
qSOFA	Quick Sequential Organ Failure Assessment
MREMS	Modified Rapid Emergency Medicine Score
NLR	Neutrophil-to-lymphocyte ratio
OR	Odds ratio
RPAS	Rapid Acute Physiology Score
RT-PCR	Real-Time Polymerase Chain Reaction
SARS-CoV-2	Severe acute respiratory syndrome coronavirus 2
SD	Standard deviation
TPRS	True positive results

## Appendix A

**Table A1:** Distribution of patients by demographic characteristics and behavioral risk factors.

N = 380		*n*	%	*p*
Gender	Female	160	42.11%	0.002
	Male	220	57.89%	
Living area	Urban	282	74.21%	<0.001
	Rural	98	25.79%	
Formal education	Uneducated	9	2.36%	<0.001
	4 years	19	5%	
	8 years	87	22.89%	
	High school	118	31.05%	
	Vocational school	92	24.21%	
	University degree	55	14.47%	
Smoking	Yes	133	35%	*p* < 0.001
	No	247	65%	
Alchool	Yes	95	25%	*p* < 0.001
	No	285	75%	
SARS-CoV-2 vaccine ≥ 1 doze	Yes	61	16%	*p* < 0.001
	No	319	84%	

**Table A2:** Biological features of hospitalized COVID-19 patients.

N = 380	Normal Values	Average ± SD	Median	Minim	Maxim	% Abnormal Values
Leucocyte	4000–10,000 μL	10764 ± 5242.83	9800	3500	53,200	30.5%
NLR	1–2	4.70 ± 4.52	0.73	0.73	44.91	56.57%
CRP	0–1 ng/dL	8.44 ± 18.14	4.6	0.5	307	94.21%
ESR	2–22 mm/30 min	52.20 ± 29.69	45	5	140	90.78%
Fibrinogen	200–400 mg/dL	665.07±	643	260	1224	96.70%
LDH	135–225 U/L	291 ± 147.68	255	98	1000	65.26%
CK	30–170 U/L	118.04 ± 148.27	134.25	20	1164	17.10%
Creatinine	0.5–1.25 mg/dL	0.93 ± 0.71	0.84	0.35	11.53	10.52%
AST	14–59 U/L	60.96 ± 46.22	46	12	458	61.31%
ALT	30–110 U/L	89.82 ± 84.68	67	10	853	73.15%
Na+	137–145 mmol/L	136.58 ± 5.09	137	114	149	30%
K+	3.5/5.1 mmol/L	4.002 ± 0.57	4.1	2.5	5.6	20.14%
Cl-	98/107 mmol/L	99.12 ± 4.42	99	78	109	34.43%
